# Underground Gold Miner Exposure to Noise, Diesel Particulate Matter and Crystalline Silica Dust

**DOI:** 10.5696/2156-9614-11.29.210301

**Published:** 2021-02-25

**Authors:** Edward K. Armah, Jeremiah A. Adedeji, Bright B. Boafo, Amma A. Opoku

**Affiliations:** 1 Department of Chemistry, Kwame Nkrumah University of Science and Technology, PMB Kumasi, Ghana; 2 Department of Chemical Engineering, Durban University of Technology, Durban, South Africa; 3 Department of Pharmacology, Kwame Nkrumah University of Science and Technology, PMB Kumasi, Ghana; 4 Department of Water and Sanitation, University of Cape Coast, Cape Coast, Ghana

**Keywords:** Exposure levels, job titles, permissible exposure levels, underground gold miners

## Abstract

**Background.:**

Respirable dust, diesel particulate matter, crystalline silica and noise pollution are the most common causes of health issues experienced by underground mine workers. Assessment of exposure levels in relation to standard regulatory body permissible levels is essential for the safety of mine workers.

**Objectives.:**

The present study compared exposure levels of diesel particulate matter, crystalline silica dust and noise experienced across different underground mine worker job titles.

**Methods.:**

Subjective sampling was employed using gravimetric air samplers over an 8-hour time weighted average for two periods designated as period 1 (first half of the year) and period 2 (second half of the year). A comparative analysis of exposure levels between job titles and in relation to the National Institute for Occupational Safety and Health (NIOSH) permissible exposure levels (PELs) was performed.

**Results.:**

In the present study, 90% of the selected job titles were over-exposed to noise and 80% were over-exposed to diesel particulate matter. The highest exposures for crystalline silica dust and diesel particulate matter were found in the 40–49-year-old age group.

**Conclusions.:**

The present study of exposure levels of diesel particulate matter, respirable dust, crystalline silica, and noise during underground gold mining demonstrates that better control mechanisms are needed to protect workers.

**Participant Consent.:**

Obtained

**Ethics Approval.:**

This study was approved by the Ethics Committee of the Kwame Nkrumah University of Science and Technology, Ghana.

**Competing Interests.:**

The authors declare no competing financial interests.

## Introduction

Mining serves as a driving economic force for countries with natural resources.^1^ In Ghana, mined natural resources include gold, manganese, bauxite, and other minerals. Because this is a lucrative sector, there has been an increase in both legal and illegal mining activities. Legal mining activities are well-controlled globally with established regulations and safety measures put in place for all workers depending on their level of exposure to toxicants. The latter remains under-recognized owing to its illegality.^2^ Although enormous revenue is generated from the mining sector in Ghana, the hazard it poses to its workers and communities in proximity to these mining fields continues to be a topic of research.^3^

Amongst all hazards, respirable dust, diesel particulate matter, crystalline silica and noise pollution remain the most common concerns owing to excavating, drilling, bogging, crushing, and hauling activities at mining sites. Risks associated with mining activities depend largely on the duration of exposure, particulate size, ventilation, and specific occupational activities. Occupational silica exposure has been described as a global health issue. Work-related silica exposure is associated with elevated mortality due to respiratory diseases such as silicosis.^4^

Epidemiological studies have linked silica exposure to elevated mortality from cardiovascular disease (CVD) as well as cancers and renal dysfunction,^5^ with statistical analyses focusing on specific CVD types producing inconsistent results. Silica is often present at industrial sites in one or more of its crystalline forms: quartz, cristobalite and tridymite.^6^ The latter two forms are known to be generated during high-temperature processes. Crystalline silica typically exists as respirable particles and silica nanoparticles, which are particles smaller than 100 nm that can enter the bloodstream. Particulates found to enter the respiratory system during breathing are divided into inhalable, thoracic, and respirable particles, which are potentially harmful to health if deposited in the lungs or airways, hence the need for stringent methods to control their exposures.^7^ Procedures for the reduction of silica inhalation based on structural, environmental, and technical improvements at underground operations have lowered the incidence of silicosis to some extent.^7^

The PEL levels for crystalline silica, respirable dust, diesel particulate matter and noise are 0.05 mg/m^3^, 5 mg/m^3^, 160 μg/m^3^ and 85 dB, respectively, over a daily eight-hour shift.^7^ The present study adopted the permissible exposure levels (PELs) according to the National Institute for Occupational Safety and Health (NIOSH).^8^ Generation of noise in mining activities continues to be an important issue, especially in underground mining.^8^

Many countries such as China, the United States, Peru, South Africa and Ghana have recognized noise as a form of pollution requiring some degree of remediation.^9^ Progressive guidelines have been developed in the past decades to reduce noise levels in underground mining operations by reducing the length of exposure to noise, monitoring the hearing of underground workers exposed to occupational noise, and encouraging hearing conservation exercises.

Diesel particulate matter (DPM) is another toxicant released during mining activities that has been found to create health concerns for underground mine workers.^9^ Diesel and petrol are the most common fuels used in mining; the latter has finer particulate matter and poses less harm in terms of deposition compared to the former. Since sulfur remains a characteristic component and pollutant in diesel fuels, the use of low-sulfur diesels, as well as diesel oxidation catalysts (DOCs) are recommended. Although these do not produce a perfect safety margin, toxicity is reduced.^10^ New diesel technologies have dramatically reduced DPM levels by 99% in developed countries, but this is not the situation in Ghana, where traditional diesel exhaust (TDE) is still used.^11^

**Abbreviations**
*DPM*: Diesel particulate matter
*NIOSH*: National Institute for Occupational Safety and Health
*PEL*: Permissible exposure level
*TWA*: Time-weighted average

Diesel particulate matter consists of carbon monoxide, carbon dioxide, hydrocarbons, oxides of nitrogen, ash, metallic abrasion particles, sulfates, and silicates. Traditional diesel exhaust systems are the main source of exposure due to partial combustion, with a minimal fraction of DPM from gaseous condensation within the exhausts. Adhering to the Occupational Safety and Health Administration's (OSHA) recommendations for DPMs could assist in preventing occupational diseases due to exposures to DPM hazards.^10^

Standards have been set forth to help limit exposure levels through monitoring and training.^7^ Diesel particulate matter is considered by the International Agency for Research on Cancer (IARC) to be a group 1 human carcinogen. The unique nature of diesel particulates, which have a large surface area able to further absorb toxins, and include mutagenic and carcinogenic compounds such as polyaromatic hydrocarbons (PAHs), increases the likelihood of carcinogenesis.^12^ Diesel particulate matter has toxic effects on the lungs, heart, kidney, placenta, brain, and liver. Respirable DPM is an emerging concern in the mining workplace, and it has been shown that individuals are exposed at a wide range of sites, ranging from domestic to industrial. Results from multiple studies in occupational sites have led regulatory bodies to put in place stringent threshold limit values (TLVs) or occupational exposure limits (OELs). The present study is a cross-sectional study evaluating diesel particulate matter, crystalline silica dust and noise exposure in various underground mining workers during two specific periods over a year.

## Methods

The underground gold mining company in the present study was chosen as a study site based on its proximity to the research institution (Kwame Nkrumah University of Science and Technology) where this project was undertaken. The mining site is located in the Ashanti Region of Ghana, specifically in Obuasi township. Worker consent was obtained, along with approval by the management of the company prior to beginning the study. Ethics approval was obtained from the Ethics Committee of the Kwame Nkrumah University of Science and Technology, Ghana.

### Sampling and data collection

Purposive sampling was employed for selecting workers to participate in the present study. This method was necessary because it focused on the underground workers of interest due to the extent of exposure levels reported by the mining site in previous years. The sampling was carried out once every two weeks over a period of 12 months and 258 workers from ten different job titles ranging from 20–59 years of age were selected for the study sample. Previous studies have shown that the effects of over-exposures to crystalline dust and silica in mine workers is predominant in those above 40 years of age.^5,7^ Therefore, it was important to determine the age ranges of the job titles in the present study to examine associations with the severity of exposure. The first sampling period was from January to June, 2016 and the second sampling period was from July to December, 2016. These two periods were chosen based on the academic calendar of the institution and to compare the effects of seasonal variations in weather. Period 1 was characterized by cold fronts (Harmattan/winter) and period 2 occurred during the hot season (summer). Job titles were selected according to level of exposure during underground operations. Personal protective equipment (PPE) was assigned to each worker before the start of their shift.

### Analysis of samples

Four (4) categories of exposures were considered in the present study: noise, diesel particulate matter, crystalline silica, and respirable dust. Gravimetric air samplers (sampling train) were made up of 10 mm nylon cyclone, pre-weighed polyvinyl chloride (PVC) cassette, clipper and connecting tube according to a previously published study.^10^ A charged constant flow pump AirChek XR5000 model 210–5000 from SKC Laboratories) calibrated to 1.7 liters per minute was used. A 37-mm diameter low-ash PVC filter cassette was used for collecting dust samples, and quartz-fiber filters were used for collecting DPM samples. Gravimetric pumps were mounted on the chest of underground workers to collect particles of respirable dust from the working area. Participants worked for eight hours, after which the gravimetric pumps were collected and analyzed for respirable dust and crystalline silica contents using a gravimetric dust sampler and an x-ray diffractometer, respectively. This was achieved as the filter holder was attached in the workers' breathing zone and the pump was also attached to the worker's belt. Gravimetric analysis of respirable dust was carried out using a 5-digit ‘TARE' BDH electronic analytical balance (precision: ± 0.01 mg). All field filters with controls were weighed at the end of each sampling day. The twelve field silver membrane filters and blank filters (four) were analyzed for crystalline silica (quartz).

The bagged dust and DPM samples were transported to an accredited laboratory for analysis. The NIOSH analytical method 7500 (Chapter Q) was used for analysis of silica and dust samples and analytical method 5040 by NIOSH reported by Pleban *et al.*^13^ was used for analysis of DPM. The requisite quality control and quality assurance systems were employed for analytical methods. The OSHA PEL standards were used as the references for the study.^10^ Noise measurements were taken 1.0–2.0 m above the floor level and 3.5 m away from sound reflecting structures (same day the measurements for the silica, DPM and dust were taken) at the target zones using the American National Standards Institute (ANSI) Type 2 sound level meter and dosimeter.^13,14,15^

The survey results were then transformed to the equivalent noise levels. The workers were categorized according to the following job titles and descriptions *(Table 1).*

**Table 1:** Summary of Job Descriptions by Job Title

**Job title**	**Job description**
Truck operator	Haul waste and ore from underground^6^
Shotcrete operator	Spray tunnels with cementing substances to hold metal mesh and rock mass together and prevent collapse^7^
Diamond operator	General drilling activities^6^
Solo operator	Drill production and service holes^11^
Jumbo operator	Uses mesh to support caved rock mass and drilling during development phase^7^,^10^
Bogger operator	Clean development and production headings off ore and/or waste by loading into trucks^10^
Cubex operator	Drill service holes and production of slots^7^,^10^
Blastmen	Charge explosives in development phase and ensure re-entry^10^
Service men	Hang ventilation fans, extend water and air lines, and other utility maintenance activities during underground operations^7^,^10^
Supervisor	Monitor and oversee underground work activities^7^,^10^

## Results

The distribution of job titles by study period (1 and 2) are presented in Table 2.

**Table 2:** Distribution of Job Titles by Study Period

**Job titles**	**Period 1**	**Period 2**
Truck operator	20	15
Shotcrete operator	45	30
Diamond operator	40	40
Solo operator	30	35
Jumbo operator	25	21
Grouters	21	15
Cubex operators	18	16
Blastmen	15	14
Service men	22	20
Inspector	22	20

**TOTAL**	**258**	**226**

**Figure 1:**
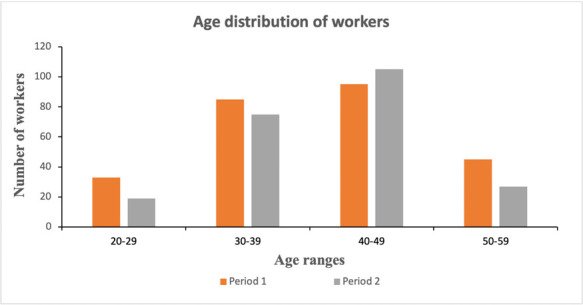
Age distribution among the participating mine workers (n = 258) over the 1-year study period

Among the 258 workers for period 1, 13% were between 20–29 years of age, 33% between 30–39 years of age, 37% between 40–49 years of age and 17% between 50–59 years of age *(Figure 1).*

Among the 226 workers for period 2, 8% were between 20–29 years of age, 33% between 30–39 years of age, 46% between 40–49 years of age and 12% between 50–59 years of age (*Figure 1*).

### Noise exposures

**Figure 2:**
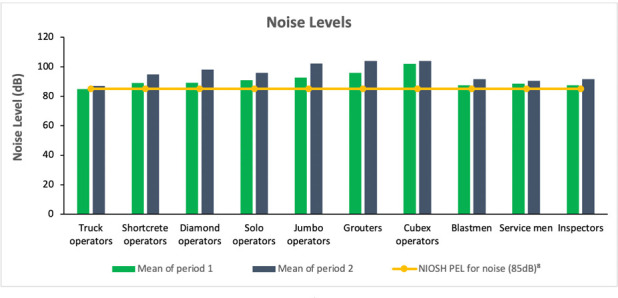
Noise exposure by mine worker job title (n=258)

All the job titles had values above the NIOSH PEL of 85 decibels (dB)^8^ during periods 1 and 2 except for truck operators for period 1 with a mean level of 84.90 dB. The highest mean level of 101.90 dB for period 1 was observed for cubex operators and in the case of period 2, the maximum mean level of 103.9 dB was observed for both cubex operators and grouters (*Figure 2*).

### Respirable dust exposure

**Figure 3:**
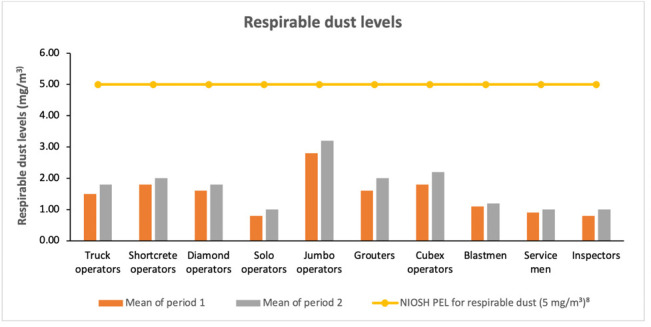
Respirable dust exposure levels by mine worker job title

In the present study, none of the job titles reported respirable dust above the permissible limit of 5.0 mg/m^3^. The highest dust exposure level was seen in jumbo operators during periods 1 and 2 with average values of 2.80 mg/m^3^ and 3.20 mg/m^3^, respectively. Cubex operators had the second highest mean exposure of 2.20 mg/m^3^ during period 2. The lowest values were reported for both solo operators and inspectors, at 0.80 mg/m^3^ and 1.00 mg/m^3^, respectively, as shown in Figure 3.

### Crystalline silica exposure

**Figure 4:**
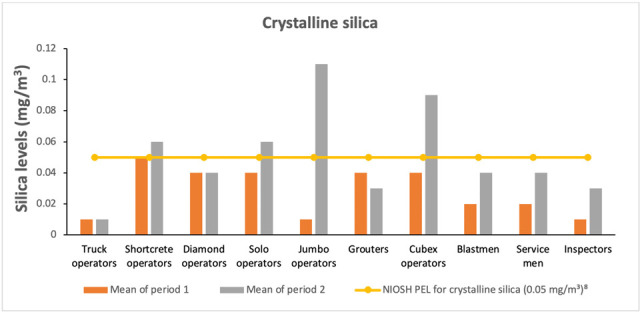
Crystalline silica exposure levels by mine worker job title

It was observed that jumbo operators recorded the highest mean exposure concentration of 0.11 mg/m^3^, representing 122% over than the NIOSH PEL, followed by cubex operators with a mean level of 0.09 mg/m^3^. The lowest mean exposure level of 0.01 mg/m^3^ was recorded for truck operators. Over-exposures of crystalline silica were observed among the shortcrete operators, jumbo operators and cubex operators with mean exposure level of 0.06 mg/m^3^, 0.11 mg/m^3^ and 0.09 mg/m^3^, respectively, which were higher than the NIOSH PEL of 0.05 mg/m^3^ *(Figure 4).*

### Diesel particulate matter exposures

**Figure 5:**
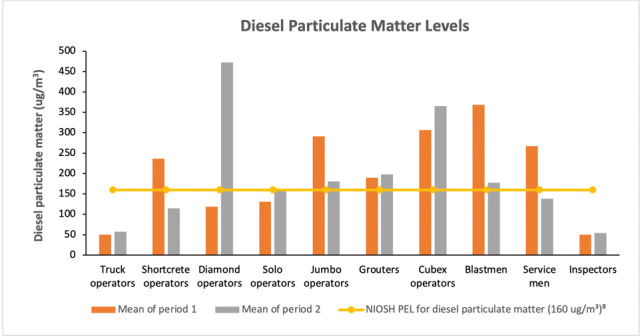
Diesel particulate matter exposure levels by mine worker job title

The NIOSH PEL for DPM over an 8-hour time-weighted average (TWA) is 160.00 μg/m^3^ was employed in this study.^8^ Diamond operators reported the highest mean level of 472.60 μg/m^3^ during period 2, followed by cubex operators with an average concentration of 364.90 μg/m^3^ for the same period. The lowest concentration was reported at 50.00 μg/m^3^ for truck operators and over-exposure to DPM was observed in shortcrete operators, diamond operators, solo operators, jumbo operators, grouters, cubex operators, blastmen and service men. Only truck operators and inspectors consistently had levels of DPM below the NIOSH PEL as shown in Figure 5. Analysis of variance (ANOVA) showed a significant difference of p<0.05 (5%), 95% confidence level for DPM exposure to the job titles.

## Discussion

The TWA limit is the airborne concentration to which nearly all mine workers may be repeatedly exposed in a normal 8-hour work shift (or a 40 hour work week).^7^ This is expressed either in parts of vapor or gas per million parts of polluted air by volume at 25°C and 101.3 kPa pressure or in milligrams of pollutant per cubic meter of air (mg/m^3^), respectively.

Silicosis is an important health problem globally, because of its potential to cause physical disability. Silicosis has a long latency period and can occur 10 to 20 years after exposure. Higher exposure to silica dust is associated with short latency and fast progression of the disease^16^ as existing PELs for crystalline silica in most countries (such as China and Canada) have been found to be inadequate in protecting miners from death due to heart disease.^17^ Previous studies according to Knight *et al.*^18^ have reported an association between the development of silicosis due to mining exposures of 0.051 mg/m^3^ - 0.075 mg/m^3^ during up two decades of work. Comparatively, in the present study, the highest mean silica value was found to be 0.11 mg/m^3^ (more than twice the PEL level of 0.05 mg/m^3^) for jumbo operators during period 2. No significant differences were observed in exposure levels between periods 1 and 2 for the reported crystalline silica concentrations among the job titles. Our previous research reported that large amounts of silica bearing rocks are broken down during underground mining operations, resulting in significant exposures for workers.^7,10^

The highest exposure levels in terms of respirable dust were found to be 2.80 mg/m^3^ and 3.20 mg/m^3^ among the jumbo operators for periods 1 and 2, respectively. This reported value could pose greater health risks to these workers although the mean values were below the PEL as respirable dust includes crystalline silica components which could cause silicosis among miners. The inspectors had the least value owing to the fact that they are not directly exposed to mining dust to a large degree. Respirable dust particles are generally considered to be a main cause of pneumoconiosis, commonly called silicosis.^19^ Most notable among them are dust particles smaller than 7 μm which are considered to be even more hazardous to workers.

For DPM exposure measurements, the highest mean level of 472.60 μg/m^3^ was reported for diamond operators during period 2, followed by blastmen with a mean value of 368.90 μg/m^3^ during period 1. The least exposure level was reported for the inspectors at 50.20 μg/m^3^, also during period 1. It was not surprising to observe very low levels of exposures recorded for the inspectors with regards to DPM since they are not directly exposed during mining operations. The higher values reported during period 1 compared to period 2 indicate the effectiveness of the control measures that were adapted after analysis of the samples in period 1. Some of these control measures involved engineering and administrative actions that were employed prior to the start of period 2. Mean concentration values for truck operators were found to be far below the PEL of 160 μg/m^3^ for periods 1 and 2, indicating low exposures of DPM. Jumbo operators, grouters, cubex operators and blastmen reported mean concentration values above the PEL for both periods 1 and 2 *(Figure 5).* This suggests that these occupations are exposed to larger amounts of diesel particulates. Diamond operators and solo operators reported DPM levels over the PEL during period 2 only. Sheesley *et al.*^20^ reported that significant health effects have been associated with DPM exposures in highly impacted micro-environments such as the mining sector. In their study, the severity of these particulates to the health of mine workers was associated with cigarette smoke and unapportioned organic carbon, which were not considered in the present study.

Prolonged exposure to higher levels of noise could result in permanent and irreversible hearing damage according to Alimohammadi *et al.*,^21^ as mining activities such as blasting, drilling, crushing and other mining and mineral processing plants are inherently noisy. Pradhan^15^ stated that mining noise is usually generated by heavy moving machinery with the highest noise level of 117dB reported by hole drillers. However, in the present study, mean noise levels were found to be above the PEL (85 dB) during periods 1 and 2 for shortcrete operators, diamond operators, solo operators, jumbo operators, grouters, cubex operators, blastmen, service men and inspectors. Truck operators reported a mean noise level of 84.99 dB just below the PEL during period 1. This indicates that hauling activities carried out by truck operators generate less noise, with lower exposures for these workers. Almost all the selected job titles (except truck operators) were exposed to noise levels above the PEL. Stringent measures were immediately suggested for these job titles to avoid further hearing impairment.

Önder^22^ investigated noise exposure levels for underground miners over a period of three years (2004–2007) and reported a maximum exposure level of 81.07 dB which is below the PEL. That study recommended modification, maintenance or replacement of noisy machines and acoustic isolation to reduce noise exposures. Two major exposure reduction methods that have gained increasing recognition in the past decades are engineering noise controls (ENC) which isolate the noise from the worker and administrative noise controls (ANC) where the worker is isolated from the noise.^8^ In effect, ENC reduces the sound level that could reach the worker and ANC reduces the time the worker is exposed to the sound level. Sensogut^23^ also indicated that personal hearing protection devices such as earplugs and earmuffs are low cost and simple noise mitigation devices, yet most miners refuse to wear them since they feel uncomfortable (self report). Other factors include the assertion that these devices are annoying or prevent them from perceiving signals such as sounds that precede a roof fall.^24^ Pradhan^15^ also identified some ANCs such as monitoring employees by initial audiogram and regular repeats and the use of warning signs.

In addition, ENC measures include placing machines on a stable foundation and where possible, using an elastic separation such as rubber blocks or steel springs and an adequate air conditioning system during mining activities to enable doors to be fully closed. These noise control measures were presented as recommendations to the mining company under which this present study was carried out.

Pawlaczyk-Luszczyńska *et al*.^25^ reported an age range of 26–55 years during noise detection for underground operations of mine workers. In the same study, it was found that noise exposures to underground workers were independent of age which is similar to results obtained from this present study. In addition, Keramydas^26^ reported in a similar study that the number of mine workers with hearing impairment increased exponentially with age until age 50, at which point 90% of miners had a hearing impairment, significantly associated with noise exposures above the PEL.

Stringent measures were immediately suggested for underground miners to reduce and avoid over-exposures to noise, DPM, dust and silica. Some of these measures include allowing job titles found to have significant exposures during underground operations in the present study to rotate shifts for surface mining activities and strict use of PPE.

The biggest limitations of the present study were that the authors did not determine maximum and minimum values for each worker to determine over-exposure. However, the mean for each job title and exposure level was selected as the preferred tool for this comparative study since the sampling size was large. In addition, the reduction in the number of workers sampled during period 2 could possibly affect this comparative study. The present study examined the extent of exposure of mine workers to DPMs, noise, silica and respirable dust in Ghana and based on the results, it is strongly recommended that an in-depth nationwide study (baseline) be conducted to determine the full extent of mining exposures in Ghana. Finally, a further limitation is that all subjects were male. This is because most underground workers are male, especially in these selected job titles.

## Conclusions

Ninety percent (90%) of the job titles in the present study were found to be over-exposed to noise, which could lead to nervousness, fatigue and hearing impairment. Higher DPMs were found to be associated with higher diesel fume exposures which are predominantly generated during underground mining operations with 80% of job titles over-exposed. Short-term exposures to higher concentrations of DPM causes headache, irritation of the eyes, nose and throat and prolonged exposures could lead to higher risk of cardiovascular disease. Short-term and long-term over-exposures of crystalline silica and respirable dust have been found to lead to pneumoconiosis, an occupational lung disease. The findings showed that most of these selected job titles are over-exposed to noise, DPM, crystalline silica and respirable dust above the NIOSH PEL for those between the age range of 40–49 years. It is recommended that engineering and administrative control measures be implemented at mining sites for these workers.

## References

[1] Liu H, Tang Z, Yang Y (2009). Identification and classification of high risk groups for Coal Workers' Pneumoconiosis using an artificial neural network based on occupational histories: a retrospective cohort study. BMC Public Health.

[2] Bio F, Sadhra S, Jackson C (2007). Respiratory symptoms and lung function impairment in underground gold miners in Ghana. Ghana Med J.

[3] Wang W, Liu H, Dai X (2015). p53/PUMA expression in human pulmonary fibroblasts mediates cell activation and migration in silicosis. Sci Rep.

[4] Bansah K, Yalley A, Dumakor-Dupey N (2016). The hazardous nature of small scale underground mining in Ghana. J Sust Min.

[5] Rees D, Murray J, Chan-Yeung M Silica, silicosis and tuberculosis [State of the Art Series. Occupational lung disease in high-and low-income countries. https://pubmed.ncbi.nlm.nih.gov/17439668/.

[6] Edwards A, Dekker J, Franz R (2011). Profiles of noise exposure levels in South African mining. J S Afri I Min Metall.

[7] Kwaansa-Ansah EE, Armah EK, Opoku F (2017). Level of Exposure of Occupational Respirable Dust to Underground Gold Mine Workers in Ghana. Glob Environ Health Saf.

[8] The National Institute for Occupational Safety and Health (NIOSH) Work related lung disease surveillance report, 2018. Centers for Disease Control and Prevention.

[9] Kjellstrom T, Lodh M, McMichael T, Ranmuthugala G, Shrestha R, Kingsland S Air, and water pollution: burden and strategies for control. In Disease Control Priorities in Developing Countries.

[10] Mensah MK, Mensah-Darkwa K, Drebenstedt C (2020). Occupational Respirable Mine Dust and Diesel Particulate Matter Hazard Assessment in an Underground Gold Mine in Ghana. JH&P.

[11] Bansah KJ, Dumakor-Dupey NK, Assan BA, Bekui P (2018). Socioeconomic and Environmental Assessment of informal artisanal and small-scale mining in Ghana. J Clean Prod.

[12] McLaughlin ML Evaluation of a Portable Chamber for Field Calibration of Particulate Matter Monitors. https://search.proquest.com/openview/9348619d2acea46e9b321c25c11758c2/1?pq-origsite=gscholar&cbl=18750&diss=y.

[13] Pleban D, Piechowicz J, Kosała K (2013). The inversion method in measuring noise emitted by machines in opencast mines of rock material. Int J Occup Saf Ergon.

[14] Donoghue A (2004). Occupational health hazards in mining: an overview. Occup Med.

[15] Pradhan C Noise survey and noise modelling of open cast machineries in mines. http://ethesis.nitrkl.ac.in/6285/.

[16] Liu Y, Rong Y, Steenland K (2014). Long-term exposure to crystalline silica and risk of heart disease mortality. Epidemiology.

[17] Mohamed SH, El-Ansary AL, Abd El-Aziz EM (2018). Determination of crystalline silica in respirable dust upon occupational exposure for Egyptian workers. Ind Health.

[18] Knight D, Ehrlich R, Cois A (2020). Predictors of silicosis and variation in prevalence across mines among employed gold miners in South Africa. BMC Public Health.

[19] Lee K-H, Jung H-J, Shin J-A (2016). Characteristics of Respirable Elemental Carbon (EC) Exposures of Household Waste Collectors. Aerosol Air Qual Res.

[20] Sheesley RJ, Schauer JJ, Smith TJ (2008). Assessment of diesel particulate matter exposure in the workplace: freight terminals. J Environ Monit.

[21] Alimohammadi I, Sandrock S, Gohari MR (2013). The effects of low frequency noise on mental performance and annoyance. Environ Monit Assess.

[22] Önder S, Önder M (2018). Statistical investigation of the noise levels in coal mining industry. J Esogu Eng Arc Fac D.

[23] Sensogut C (2007). Occupational noise in mines and its control-A case study. Pol J Environ Stud.

[24] Chadambuka A, Mususa F, Muteti S (2013). Prevalence of noise induced hearing loss among employees at a mining industry in Zimbabwe. Afr Health Sci.

[25] Pawłaczyk-Luszczyńska M, Dudarewicz A, Waszkowska M (2003). Assessment of annoyance from low frequency and broadband noises. Int J Occup Environ Health.

[26] Keramydas D, Bakakos P, Alchanatis M (2020). Investigation of the health effects on workers exposed to respirable crystalline silica during outdoor and underground construction projects. Exp Ther Med.

